# Vaginal *PRA1* identifies clinically relevant *Candida albicans* vaginitis and excludes asymptomatic colonization: an exploratory study

**DOI:** 10.1186/s12866-026-05405-w

**Published:** 2026-07-17

**Authors:** Elena Roselletti, Barbara Kozma, David Ratonyi, Alexandra Kunkli, Bence Kozma, Peter Takacs, Duncan Wilson

**Affiliations:** 1 Medical Research Council Centre for Medical Mycology, The University of Exeter, University of Exeter, Geoffrey Pope Building Stocker Road, Exeter, UK; 2 Department of Obstetrics and Gynecology, Faculty of Medicine, University of Debrecen, Debrecen, Hungary; 3 Division of Female Pelvic Medicine and Reconstructive Surgery, Department of Obstetrics and Gynecology, Eastern Virginia Medical School, Norfolk, VA USA

**Keywords:** *Candida albicans*, *PRA1*, Vaginitis, Vulvovaginal candidiasis, Biomarker

## Abstract

**Background:**

Accurate interpretation of *Candida* detection in women with vulvovaginal symptoms remains difficult, because organism recovery may reflect either clinically relevant infection or nonpathogenic colonization. We have recently demonstrated that the secreted fungal zinc-binding protein, Pra1 is responsible for driving the inflammatory immunopathology of VVC. We therefore reasoned that *PRA1* expression by *Candida albicans* in the vagina may act as a diagnostic tool to discriminate between commensal colonisation and active disease.

**Methods:**

We conducted a 12-week prospective, single-center exploratory biomarker analysis nested within a registered parent clinical trial. Nine women with vulvovaginal symptoms provided cervicovaginal lavage samples and clinical data at baseline and at weeks 4, 8, and 12. This report does not present the primary efficacy analysis of the randomized trial. At each visit, *Candida* culture status and clinical severity score (CSS) were recorded. Symptomatic *Candida* vaginitis was defined as *Candida* culture positivity with CSS ≥ 3 at the same visit. Species identification of fungal isolates was performed by MALDI-TOF mass spectrometry, and vaginal *PRA1* was quantified by SYBR Green real-time qPCR. Vaginal *PRA1* concentrations were assessed as a time-varying same-visit biomarker using repeated-measures approaches, non-parametric comparisons, correlation analyses, and receiver operating characteristic (ROC) curves.

**Results:**

*PRA1* concentrations were substantially higher in *Candida* culture-positive than in culture-negative samples (median 4,011 vs. 21.5 pg/µL, *p* = 3.99 × 10⁻⁵). *PRA1* was also higher in samples meeting criteria for symptomatic *Candida* vaginitis than in asymptomatic culture-negative samples (median 11,762 vs. 21.5 pg/µL, *p* = 8.15 × 10⁻⁵). Among culture-positive observations, *PRA1* was associated with symptom severity (Spearman rho = 0.67; Pearson *r* = 0.56 after log10 transformation). Same-visit discrimination was high in this cohort for both *Candida* culture positivity and symptomatic *Candida* vaginitis (AUC approximately 0.95 for each outcome). In this small exploratory cohort, lower *PRA1* thresholds achieved 100% sensitivity and negative predictive value for clinically significant *Candida* vaginitis. These estimates require validation in larger studies.

**Conclusions:**

These preliminary findings suggest that vaginal *PRA1* may help distinguish symptomatic *Candida*-associated disease from clinically insignificant *Candida* detection and may be particularly useful as a rule-out biomarker. Given the small sample size and exploratory design, these results should be regarded as hypothesis-generating and require validation in larger, independent cohorts. The study protocol was approved by the Hungarian National Institutional Review Medical Research Council (approval No. 3282-7/2023/EUIG).

The parent trial is registered at ClinicalTrials.gov (NCT05895162; 2023-06-08).

**Supplementary Information:**

The online version contains supplementary material available at https://doi.org/10.1186/s12866-026-05405-w.

## Introduction

Vulvovaginal candidiasis is a common cause of vulvovaginal symptoms and an important clinical and public-health problem in women of reproductive age. A persistent diagnostic difficulty is that detecting *Candida* does not, by itself, establish that the symptoms are caused by *Candida albicans.* Asymptomatic carriage is common, so culture or molecular detection must be interpreted together with clinical findings rather than as an isolated marker of disease [1, 2]. The burden of VVC and recurrent VVC remains substantial, and *C. albicans* accounts for a large proportion of mucosal Candida infections worldwide [3]. *C. albicans* pathogenicity involves multiple processes, including epithelial adherence, morphologic switching, host adaptation, and immune interactions; adhesin families, such as agglutinin-like sequence proteins, contribute to colonization and host-cell engagement [4, 5]. At the same time, the increasing complexity of antifungal management, including concerns about resistance and non-*albicans* species, has strengthened the need for accurate species-level diagnosis using microscopy, culture, susceptibility testing, or molecular approaches when clinically indicated [6]. Molecular and genotyping methods have further improved epidemiologic characterization of *Candida* isolates, but organism identification alone does not necessarily indicate active symptomatic infection [7]. At present, no clinically validated biomarker reliably distinguishes active *Candida*-associated disease from asymptomatic colonization, which underscores the need for objective markers that reflect fungal activity rather than organism presence alone.

Pra-1 (pH-regulated antigen 1) is a *C. albicans* protein that has been studied in relation to host interaction, complement binding, zinc acquisition, and inflammatory signaling. The protein is present at the fungal surface and can also be released extracellularly, allowing potential detection in cervicovaginal secretions [8]. Molecular genetic studies have shown that PRA1 Pra1 acts as a “zincophore”, capturing zinc from host cells and, as such, the PRA1 gene is transcriptionally unregulated in response to limitation of this essential trace mineral [9]. Prior translational work, including studies of human vaginal samples and experimental models, identified Pra1 as an important mediator of inflammatory immunopathology in VVC [10]. *PRA1* expression directly correlated with the inflammatory cytokines IL-1β and IL-8 during clinical VVC in women. Moreover, genetic deletion of *PRA1* in *C. albicans* blocked recognition by human neutrophils in an ex vivo transmigration model and prevented inflammation in a mouse model of VVC. These observations raise a clinically distinct question: whether vaginal *PRA1* measurement can help separate symptomatic *Candida* vaginitis from *Candida* detection that is not clinically relevant. We therefore hypothesized that vaginal *PRA1* levels would be higher in clinically defined symptomatic *Candida* vaginitis than in asymptomatic *Candida* detection, and we evaluated *PRA1* as a non-invasive, same-visit biomarker capable of distinguishing clinically relevant infection from colonization.

## Materials and methods

### Study design and participants

We performed a prospective, single-center longitudinal study nested within an ongoing randomized clinical trial at the outpatient clinic of the Department of Obstetrics and Gynecology, University of Debrecen, Hungary. The present report constitutes a preliminary exploratory analysis of consecutively enrolled participants evaluated between June 2023 and April 2024. The study protocol was approved by the Hungarian National Institutional Review Medical Research Council (approval No. 3282-7/2023/EUIG), and written informed consent was obtained from all participants prior to any study-related procedure. Women aged ≥ 18 years presenting with vulvovaginal complaints (including discharge, malodor, pruritus, pain, dysuria, skin irritation, burning, dyspareunia, or related symptoms) were eligible for inclusion. Exclusion criteria comprised pregnancy, breastfeeding, abnormal cervical cytology within the preceding year, gynecologic malignancy, relevant systemic disease, known azole hypersensitivity, liver disease, recent systemic or topical antifungal treatment or systemic antibiotic use within a predefined washout period, and any laboratory-confirmed sexually transmitted infection. For the purposes of the current analysis, data from nine women were included and assessed at four predefined time points: baseline (week 0), week 4, week 8, and week 12. At each visit, demographic and clinical data as well as interval history were recorded, including age, body mass index (BMI), parity, history of recurrent infections, and interim treatments.

### Sample collection

Gynecologic examinations were conducted using a plastic speculum and plastic instruments to minimize exogenous metal contamination while documenting clinical signs such as discharge and mucosal irritation. Following symptom assessment, cervicovaginal lavage (CVL) samples were collected at each visit. For CVL sampling, 10 mL sterile normal saline was instilled onto the vaginal walls and cervix and left in contact for 60 s, followed by three consecutive aspiration–reinstillation cycles using a plastic syringe; the pooled fluid was then aspirated from the posterior fornix. Samples were aliquoted and stored at − 80 °C until analysis. Vaginitis status was evaluated at each time point by an experienced obstetrician–gynecologist based on symptom assessment, gynecologic examination, and routine vaginal culture performed according to institutional standards. When vaginal yeast infection was identified, participants received oral antifungal therapy according to local clinical practice, and additional treatment for other vaginal conditions was initiated when clinically indicated; all interim therapies were documented at follow-up visits. As part of the ongoing randomized clinical trial (ClinicalTrials.gov identifier: NCT05895162; registered June 8, 2023), participants in the symptomatic cohort were randomized 1:1. The control arm received standard treatment consisting of a single oral dose of fluconazole at baseline, whereas the intervention arm received fluconazole followed by intravaginal JUVIA zinc-containing hydrogel (Fempharma LLC, Debrecen, Hungary). Participants assigned to the intervention arm were instructed to self-administer 2 mL vaginal gel using the provided applicator for 12 weeks (daily during the first 2 weeks, then twice weekly for the subsequent 10 weeks). Adherence was monitored using participant logs, and adverse events were actively queried and recorded at each visit.

The present manuscript is limited to an exploratory *PRA1* biomarker analysis and does not evaluate the primary clinical efficacy endpoint of the registered trial. For this analysis, vaginal *PRA1* concentrations, *Candida* culture status, and clinical severity scores were analyzed at the visit level. The primary outcomes of the present report were same-visit *Candida* culture positivity and symptomatic *Candida* vaginitis, defined as concurrent *Candida* culture positivity and CSS ≥ 3. Because the objective was biomarker discrimination rather than treatment efficacy, the analysis was primarily restricted to participants who did not receive intravaginal JUVIA hydrogel. Relevant CONSORT reporting items were addressed where applicable, and a completed CONSORT checklist is provided as an additional file. Of 102 women assessed for the parent trial, 54 were enrolled into the symptomatic cohort; 22 were allocated to the control arm, and 9 of these had paired *PRA1* and culture data available and constitute the present analytic subset. This availability-based, control-arm subset was used so that biomarker discrimination could be evaluated independently of the zinc-hydrogel intervention; its potential for selection bias is acknowledged in the Limitations.

### Outcome definitions

For the present analysis, a priori operational definitions were established based on the Candida Severity Score (CSS) [11] and routine vaginal culture results. All classifications were applied at the visit level (same-visit). A visit was classified as symptomatic *Candida* vaginitis when CSS ≥ 3 occurred together with a positive vaginal culture, and as asymptomatic culture-negative when CSS < 3 occurred together with a negative vaginal culture. The CSS was applied as described in reference 10 using the ≥ 3 threshold; the item-level composition of the score follows that source and was not separately enumerated in this report.

### Microbiological culture and species identification

Vaginal samples were cultured on blood agar (BA), eosin methylene blue (EMB) agar, and Sabouraud agar (SAB). Microbiological reports also included a statement on the presence and characteristics of the normal vaginal flora. Fungal isolates, including scant growth, were identified to species level, and routine antifungal susceptibility testing was performed and reported in accordance with institutional laboratory protocols. The extent of fungal growth on Sabouraud agar was additionally recorded (semiquantitatively and/or by colony count, as applicable). Species identification was carried out by matrix-assisted laser desorption/ionization time-of-flight mass spectrometry (MALDI-TOF MS) using Bruker microflex LRF.

### RNA extraction and *PRA1* quantification by qPCR

For RNA analysis, samples were pelleted directly without dilution, resuspended in extraction buffer, and the RNA extracted directly from the pellet, so that PRA1 was quantified from the recovered cellular material rather than from diluted lavage fluid. Fungal RNA was extracted following the instructions of RiboPure - Yeast Kit (MilliporeSigma, Burlington, Massachusetts, USA). Following DNase treatment, RNA integrity and concentration was confirmed using a NanoDrop spectrophotometer and 500ng RNA from each sample was reverse transcribed to cDNA using RevertAid first strand cDNA synthesis kit. To evaluate gene expression, a real-time qPCR (QuantStudio 7 pro, Applied Biosystems, Thermo Fisher Scientific, Waltham, Massachusetts, USA) was performed using SYBR Green (Power up Sybr Green master mix). For real-time PCR reaction, 1 µl of 500 ng of cDNA was used for each sample and each gene. All the primers used are reported in PMID: 38,055,800. The quantity of RNA per µl for each sample was calculated using a standard curve made with an *in vitro C. albicans* culture. This standard curve was generated from NanoDrop-defined concentrations of PRA1 derived from an in vitro Candida albicans culture; the Ct values of the clinical samples were plotted against these known PRA1 concentrations. Reported values (pg/µL) therefore reflect the actual quantity of PRA1 transcript rather than total RNA. Standard controls, including no-template (water) controls, were included in every run. For each sample, expression of the housekeeping gene ACT1 (encoding actin) was also assessed as an internal positive control and proxy for fungal load; ACT1 data are not reported here, as the aim was to evaluate PRA1 as a disease biomarker rather than to quantify fungal burden. This study used targeted qPCR quantification only; no 16 S rRNA or shotgun sequencing, OTU/ASV generation, read filtering, or taxonomic-database assignment was performed. Because the analyzed material consisted of clinical specimens obtained from individual trial participants, biological replicates in the conventional laboratory sense were not applicable; each measurement corresponds to a distinct patient sample. All samples were analyzed in technical triplicate for qRT-PCR quantification. Because samples were pelleted and extracted directly rather than analysed as diluted lavage fluid, no correction for lavage dilution was required. Consistent with the inherent sensitivity of PCR-based detection, the assay was characterised by its calibration to a standard curve rather than by a fixed limit of detection; formal assay validation specifically for cervicovaginal lavage was beyond the scope of this exploratory study and is noted in the Limitations.

Vaginal *PRA1* concentrations (pg/µL) were measured at baseline (V0) and at three follow-up visits (V1–V3). At each visit, *Candida* (CA) culture was performed, and clinical symptoms were assessed using a standardized clinical score. Two outcomes were defined: *CA* culture positivity, and symptomatic *Candida* vaginitis, defined as concurrent *CA* culture positivity and a CSS ≥ 3 at the same visit.

### Statistical analysis

*PRA1* was analyzed as a time‑varying biomarker for same‑visit classification. Because participants contributed repeated observations across visits, all models used a repeated‑measures framework (mixed‑effects logistic regression or generalized estimating equations, as appropriate) to account for within-participant correlation across visits. Correlations between *PRA‑1* concentrations and clinical symptom scores among *Candida* culture‑positive women were assessed using Spearman rank correlation; Pearson correlation was additionally evaluated after log₁₀ transformation of *PRA‑1* concentrations. Due to marked right‑skew, *PRA‑1* concentrations were log₁₀‑transformed for regression and ROC analyses, and non‑parametric methods were used for group comparisons. Group differences in *PRA‑1* levels were assessed using Mann–Whitney U tests. Diagnostic discrimination was evaluated with receiver operating characteristic (ROC) curves and area under the curve (AUC). Diagnostic performance was summarized at prespecified *PRA‑1* thresholds (100, 200, and 300 pg/µL), reporting sensitivity, specificity, positive predictive value, and negative predictive value. All analyses focused on contemporaneous (same‑visit) classification rather than forward prediction, and two‑sided p values < 0.05 were considered statistically significant. No sequencing-based microbiome dataset was generated, so multiple-comparison correction for such data was not applicable; a formal missing-data procedure was not specified for this exploratory analysis.

## Results

Baseline characteristics of the study cohort are summarized in Table 1. A total of 9 participants were included, with a mean (± SD) age of 34.0 ± 12.41 years and a mean body mass index of 24.38 ± 3.84 kg/m². Three participants (33.33%) reported a history of vaginal delivery, one participant (11.11%) was postmenopausal, and two participants (22.22%) had a history of prior gynecological intervention (Table 1).

**Table 1:** Baseline characteristics of the study cohort

Age (years, mean ± SD)	34 ± 12.41
BMI (kg/m2, mean ± SD)	24.38 ± 3.84
History of vaginal delivery (n, %)	3 (33.33)
Postmenopausal (n, %)	1 (11.11)
History of gynecological intervention (n, %)	2 (22.22)

*N* = 9; values are mean ± SD or n (%). Units: age in years; BMI in kg/m²

### *PRA1* levels by *Candida* culture status

**Fig. 1:**
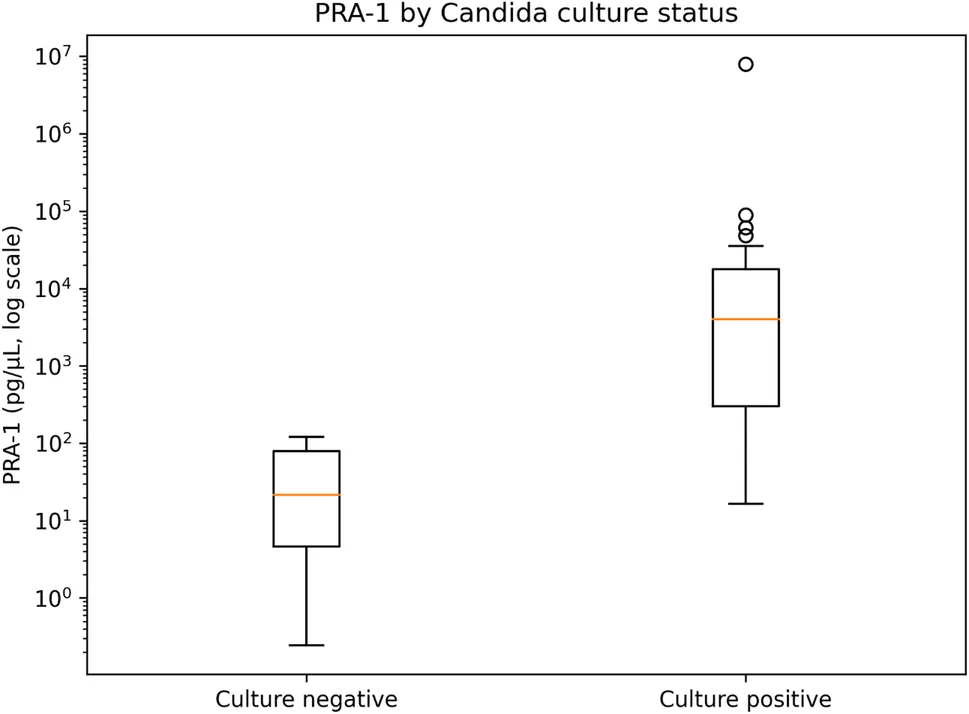
PRA-1 concentrations by *Candida* culture status. Boxplot showing vaginal PRA1 concentrations (pg/µL, log scale) stratified by Candida culture status. PRA1 levels were significantly higher in culture-positive samples

Vaginal *PRA1* concentrations were markedly higher in *Candida* culture-positive samples (*n* = 20) than in culture-negative samples (*n* = 11). Median *PRA1* concentrations were 4,011 pg/µL (IQR 299–17,636) in culture-positive samples and 21.5 pg/µL (IQR 4.6–78.8) in culture-negative samples (Mann–Whitney U test, *p* = 3.99 × 10⁻⁵). The geometric mean *PRA1* concentration was 3,340 pg/µL (95% CI 810–13,766) in culture-positive samples, compared with 13.4 pg/µL (95% CI 3.1–57.9) in culture-negative samples (Fig. 1).

### *PRA1* levels by clinical disease status

**Fig. 2:**
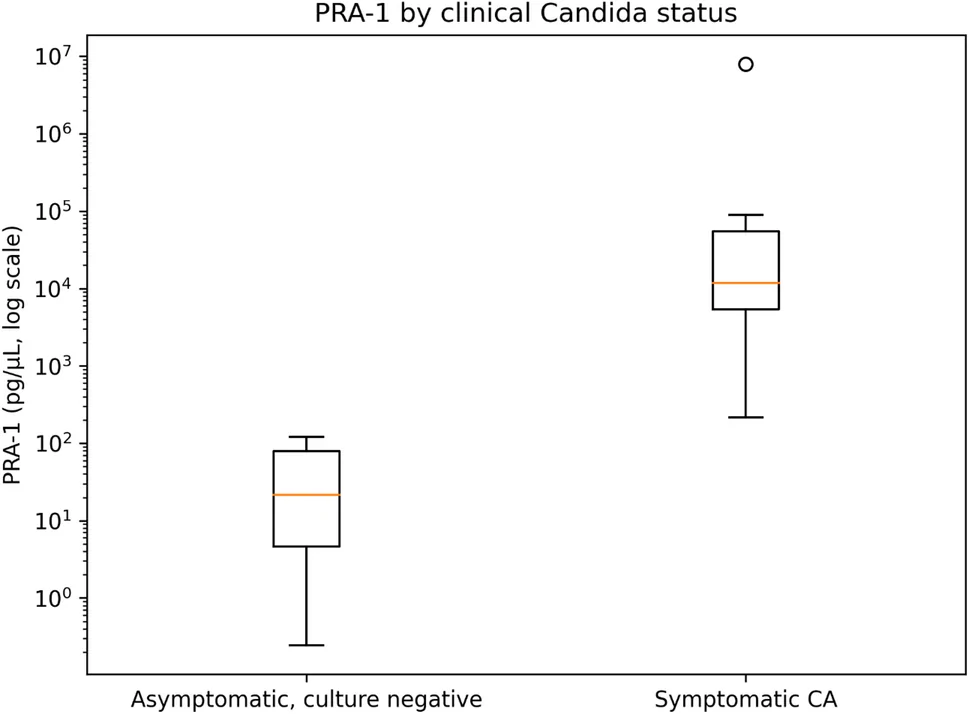
*PRA1* concentrations by clinical *Candida* status. Boxplot showing vaginal PRA1 concentrations (pg/µL, log scale) in asymptomatic culture-negative women and women with symptomatic Candida vaginitis

When clinical symptoms were incorporated, separation in *PRA1* levels was even more pronounced. Women with symptomatic Candida vaginitis (*n* = 11) had higher median *PRA1* concentrations than asymptomatic culture-negative women (*n* = 11): 11,762 pg/µL (IQR 5,373–54,911) versus 21.5 pg/µL (IQR 4.6–78.8), respectively (Mann–Whitney U test, *p* = 8.15 × 10⁻⁵). The geometric mean *PRA1* concentration was 19,815 pg/µL (95% CI 3,423–114,693) compared with 13.4 pg/µL (95% CI 3.1–57.9) in asymptomatic culture-negative women (Fig. 2).

Among *Candida* culture–positive women (*n* = 20), vaginal *PRA1* levels were significantly associated with clinical symptom severity. While no linear correlation was observed using raw *PRA1* concentrations (Pearson *r* = 0.01, *p* = 0.96), a strong monotonic association was present (Spearman ρ = 0.67, *p* = 0.001). After log10 transformation of *PRA1*, a moderate linear correlation with symptom score was observed (Pearson *r* = 0.56, *p* = 0.011).

**Fig. 3:**
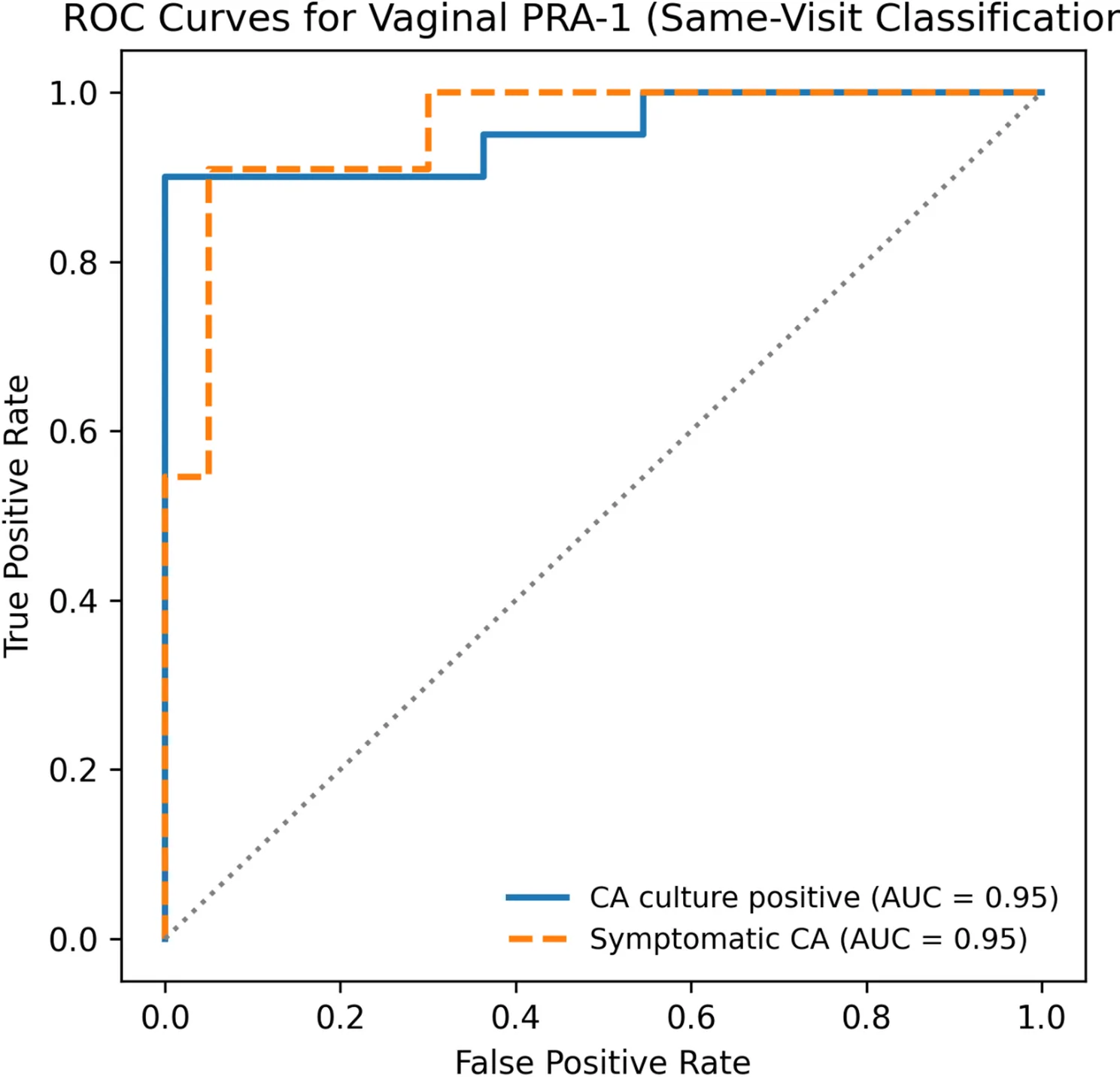
ROC curves for *PRA1* classification of *Candida* outcomes. Receiver operating characteristic (ROC) curves illustrating the same-visit discriminatory performance of vaginal PRA1 for CA culture positivity and symptomatic Candida vaginitis. AUC values indicate strong discrimination in this exploratory cohort

As shown in Table 2; Fig. 3, vaginal *PRA1* demonstrated high same-visit discrimination in this cohort for *Candida* culture positivity, with an area under the ROC curve (AUC) of approximately 0.95. Across prespecified *PRA1* thresholds, sensitivity and specificity were consistently high, indicating strong overall diagnostic performance. Lower *PRA1* cutoffs favored sensitivity, whereas higher cutoffs improved specificity, supporting the use of *PRA1* as a screening biomarker for the presence of *Candida*. Given the small cohort and repeated observations, these diagnostic-performance estimates should be interpreted as exploratory.

**Table 2:** Diagnostic performance of vaginal PRA-1 for *Candida* culture positivity (same-visit classification)

PRA1 cutoff (pg/µL)	Sensitivity	Specificity	PPV	NPV
≥ 100	0.90	0.82	0.90	0.82
≥ 200	0.90	1.00	1.00	0.85
≥ 300	0.75	1.00	1.00	0.69

When clinical relevance was incorporated by restricting the outcome to symptomatic *Candida* vaginitis (culture-positive with a clinical score ≥ 3), diagnostic performance was further refined (Table 3; Fig. 3). *PRA1* maintained high discrimination (AUC ≈ 0.95) in this cohort, with lower thresholds achieving 100% sensitivity and negative predictive value in this dataset. These findings indicate that low *PRA1* concentrations reliably exclude clinically significant *Candida* vaginitis, while higher concentrations are associated with symptomatic disease. Collectively, the ROC analyses and threshold-based performance metrics support the potential of *PRA1* as a rule-out biomarker for symptomatic *Candida* vaginitis rather than a standalone confirmatory diagnostic test, pending validation in larger studies.

**Table 3:** Diagnostic performance of vaginal PRA-1 for symptomatic *Candida* vaginitis (culture positive with clinical score ≥ 3; same-visit classification)

PRA1 cutoff (pg/µL)	Sensitivity	Specificity	PPV	NPV
≥ 100	1.00	0.55	0.55	1.00
≥ 200	1.00	0.65	0.61	1.00
≥ 300	0.91	0.75	0.67	0.94

## Discussion

This exploratory prospective analysis suggests that vaginal *PRA1* concentrations provide clinically useful information about Candida-associated vaginitis beyond culture status alone. *PRA1* concentrations were substantially higher in *Candida* culture-positive observations than in culture-negative observations, and the separation was stronger when culture positivity was combined with symptom severity to define symptomatic *Candida* vaginitis. The ROC analyses showed high same-visit discrimination in this cohort for both *Candida* culture positivity and symptomatic *Candida* vaginitis, with lower thresholds achieving 100% sensitivity and negative predictive value for clinically significant *Candida* vaginitis in this relatively small dataset.

These findings support the concept that low vaginal *PRA1* concentrations may be most clinically useful for excluding symptomatic Candida-associated disease rather than as a standalone confirmatory test.

The clinical need for such a biomarker arises from a familiar problem in vaginitis care. *Candida* organisms may be recovered from women without *Candida*-driven symptoms, and symptoms such as pruritus, burning, irritation, dyspareunia, and altered discharge are not specific to fungal infection [6, 12, 13]. Culture remains important because it confirms organism recovery and permits species identification and susceptibility testing, but it does not directly indicate whether Candida is behaving as a commensal organism or as a driver of active disease [14]. Molecular assays can improve analytic sensitivity and speed, but a positive nucleic-acid result still requires clinical interpretation, because organism presence may not be the main cause of symptoms [15]. A complementary marker reflecting fungal activity or host–pathogen interaction could therefore help place microbiological findings in a more clinically meaningful context.

PRA1 is biologically suited for investigation in this setting because it is connected to C. albicans host interaction and nutrient acquisition pathways rather than merely to organism presence. Existing literature has described *PRA1* in relation to complement regulation, zinc scavenging, epithelial interaction, and inflammatory responses [8, 15–18]. Prior work also showed that PRA1 is relevant to *C. albicans*-associated vaginal inflammation and that zinc availability can influence this pathway [16]. The present study addresses a different, diagnostic question: whether the measurable concentration of vaginal *PRA1* can function as a clinical biomarker of symptomatic disease. Our data demonstrate an association rather than a validated mechanism: *PRA1* tracked with culture positivity, symptom-defined disease, and symptom severity, but the present study did not include functional experiments to confirm the mechanistic basis of these associations. Our data are consistent with that possibility, because *PRA1* was associated with culture positivity, symptom-defined disease, and symptom severity among culture-positive samples. These results also fit with the broader understanding that *C. albicans* pathogenicity depends on regulated virulence traits and environmental adaptation rather than simple organism detection alone [19].

From a practical standpoint, the strongest potential application of *PRA1* in this dataset is rule-out testing. In symptomatic women, a low *PRA1* value could help clinicians interpret a positive or equivocal *Candida* result more cautiously and may reduce unnecessary antifungal exposure when the overall clinical picture does not support active *Candida* vaginitis. Conversely, higher *PRA1* concentrations may identify a subgroup in whom *Candida* detection is more likely to reflect clinically relevant disease. This interpretation would not replace microscopy, culture, species identification, or susceptibility testing, all of which remain important for patient management, particularly when non-albicans *Candida* species, mixed infections, recurrent disease, or treatment failure are present. Rather, *PRA1* measurement may provide an additional layer of biological interpretation that could be integrated with standard diagnostic workflows.

Several limitations should be considered. The study was a small pilot exploratory analysis including only nine women and 31 visit-level observations. Therefore, all diagnostic-performance estimates should be interpreted with substantial caution and should be regarded as preliminary and hypothesis-generating rather than confirmatory. Although repeated-measures analytical approaches were used, multiple observations from the same participants inevitably limit the precision, independence, and generalizability of the estimates. The analysis was designed to evaluate same-visit associations between vaginal *PRA1* concentrations, Candida culture status, and clinical findings. The present study therefore does not determine whether *PRA1* can predict future persistence, relapse, recurrence, treatment response, or longitudinal disease course. Third, the study was conducted at a single center and was primarily restricted to an availability-based subset of participants from the control arm of a registered parent trial. This design may introduce selection bias and limits external validity. In addition, antifungal therapy administered during follow-up may have influenced symptoms, culture status, and *PRA1* concentrations, and this should be considered when interpreting same-visit associations. Confidence intervals for AUC, sensitivity, specificity, PPV, and NPV were not available from the reported data and should be included in future validation studies. The per-sample *Candida* species distribution was not tabulated, and formal assay validation specifically for cervicovaginal lavage, including limits of detection, limits of quantification, reproducibility, and clinically applicable decision thresholds, was beyond the scope of this pilot study. The study did not include parallel metabolomic, transcriptomic, microbiome, or inflammatory-marker analyses to support mechanistic interpretation. *PRA1* was measured in cervicovaginal lavage under research conditions, and clinically practical sampling methods, assay standardization, reproducibility, and validated diagnostic cutoffs must be established before any routine clinical application can be considered. Larger, independent, prospectively designed studies are required to confirm whether vaginal *PRA1* has clinically useful diagnostic value.

Despite these limitations, the findings provide preliminary evidence that vaginal *PRA1* concentrations may help distinguish symptomatic *Candida* vaginitis from clinically insignificant *Candida* detection. Overall, *PRA1* showed promising preliminary results in this small exploratory cohort and should be validated in larger, independent studies. Larger studies should confirm the observed diagnostic performance, evaluate *PRA1* across *Candida* species and mixed vaginal infections, and determine whether *PRA1*-guided algorithms improve treatment decisions and reduce unnecessary antifungal use.

## Conclusion

In this small pilot exploratory cohort, vaginal *PRA1* concentrations were higher in visits with *Candida*-associated vaginitis than in culture-negative asymptomatic visits. *PRA1* showed preliminary same-visit discriminatory performance for symptomatic *Candida* vaginitis, with high negative predictive value at lower thresholds in this dataset. However, these findings must be interpreted with substantial caution because of the limited sample size, repeated visit-level observations, and availability-based analytic subset. Vaginal *PRA1* should therefore be considered only as a candidate biomarker at this stage. Larger, independent, prospectively designed validation studies are required to confirm its diagnostic accuracy, define clinically useful thresholds, and determine whether *PRA1*-guided testing can meaningfully improve clinical decision-making or reduce unnecessary antifungal treatment.

## Supplementary Information

Supplementary Material 1.

## Data Availability

The datasets used and/or analyzed during the current study are available from the corresponding author upon reasonable request.

## Abbreviations

ALS: Agglutinin-like sequence proteins
BMI: Body mass index
CSS: Candida Severity Score
CVL: Cervicovaginal lavage
MALDI-TOF MS: Matrix-assisted laser desorption/ionization time-of-flight mass spectrometry
PCR: Polymerase Chain Reaction
PRA1: pH-regulated antigen 1
ROC: Receiver operating characteristic
RVVC: Recurrent vulvovaginal candidiasis
Zrt1: Zinc-regulated transporter 1
Zap: zinc-responsive transcription factor

## References

[1] Parambath S, Dao A, Kim HY, Zawahir S, Izquierdo A, Tacconelli E, et al. Candida albicans-A systematic review to inform the World Health Organization Fungal Priority Pathogens List. Med Mycol. 2024;62(6):myae045. 10.1093/mmy/myae045 PMC11210619 38935906

[2] Véronique L, Véronique A, Guillaume C, Jean-Michel C, Françoise A. Candida albicans cells exhibit media specific proteomic profiles during induction of filamentation. BMC Microbiol. 2024;11–27(1):500. 10.1186/s12866-024-03627-4 PMC11600622 39592958

[3] Richardson JP. Candida albicans: A Major Fungal Pathogen of Humans. Pathogens. 2022;11(4):459. 10.3390/pathogens11040459 PMC9025087 35456133

[4] Faustino M, Ferreira CMH, Pereira AM, Carvalho AP. Candida albicans: the current status regarding vaginal infections. Appl Microbiol Biotechnol. 2025;109(1):91. 10.1007/s00253-025-13478-2 PMC11985607 40210803

[5] Nielsen S, White K, Preiss K, Peart D, Gianoulias K, Juel R, et al. Growth and Antifungal Resistance of the Pathogenic Yeast, Candida Albicans, in the Microgravity Environment of the International Space Station: An Aggregate of Multiple Flight Experiences. Life. 2021;/4(4):283. 10.3390/life11040283 PMC8067245 33801697

[6] Donders G, Sziller IO, Paavonen J, Hay P, de Seta F, Bohbot JM, et al. Management of recurrent vulvovaginal candidosis: Narrative review of the literature and European expert panel opinion. Front Cell Infect Microbiol. 2022;12:934353. 10.3389/fcimb.2022.934353 PMC9504472 36159646

[7] Tamai IA, Pakbin B, Fasaei BN. Genetic diversity and antifungal susceptibility of Candida albicans isolates from Iranian HIV-infected patients with oral candidiasis. BMC Res Notes. 2021;03–10(1):93. 10.1186/s13104-021-05498-8 PMC7945322 33691787

[8] Luo S, Blom AM, Rupp S, Hipler U, Hube B, Skerka C, et al. The pH-regulated Antigen 1 of Candida albicans Binds the Human Complement Inhibitor C4b-binding Protein and Mediates Fungal Complement Evasion. J Biol Chem. 2011;3–11(10):8021–9. 10.1074/jbc.M110.130138 PMC3048689 21212281

[9] Wilson D, Citiulo F, Hube B. Zinc exploitation by pathogenic fungi. PLoS Pathog. 2012;8(12):e1003034. 10.1371/journal.ppat.1003034 . Epub 2012 Dec 20. PMID: 23308062; PMCID: PMC3534374. PMC3534374 23308062

[10] Hefny ZA, Ji B, Elsemman IE, Nielsen J, Van Dijck P. Transcriptomic meta-analysis to identify potential antifungal targets in Candida albicans. BMC Microbiol. 2024;02–27(1):66. 10.1186/s12866-024-03213-8 PMC10898158 38413885

[11] Mehta A, Bachmann G. Vulvovaginal complaints. Clin Obstet Gynecol. 2008;–09(3):549–55. 10.1097/GRF.0b013e3181809a26 18677149

[12] Ceccarani C, Foschi C, Parolin C, D’Antuono A, Gaspari V, Consolandi C et al. Diversity of vaginal microbiome and metabolome during genital infections. Sci Rep. 2019;9(1):14095. 10.1038/s41598-019-50410-x PMC6773718 31575935

[13] Farr A, Effendy I, Frey Tirri B, Hof H, Mayser P, Petricevic L, et al. Guideline: Vulvovaginal candidosis (AWMF 015/072, level S2k). Mycoses. 2021;–06(6):583–602. 10.1111/myc.13248 PMC8248160 33529414

[14] Sobel JD. Vulvovaginal candidosis. Lancet. 2007;06–09(9577):1961–71. 10.1016/S0140-6736(07)60917-9 17560449

[15] Gaydos CA, Beqaj S, Schwebke JR, Lebed J, Smith B, Davis TE, et al. Clinical Validation of a Test for the Diagnosis of Vaginitis. Obstet Gynecol. 2017;–07(1):1–9. 10.1097/AOG.0000000000002090 PMC5635603 28594779

[16] Roselletti E, Pericolini E, Nore A, Takacs P, Kozma B, Sala A, et al. Zinc prevents vaginal candidiasis by inhibiting expression of an inflammatory fungal protein. Sci Transl Med. 2023;15(725):eadi3363. 10.1126/scitranslmed.adi3363 PMC7616067 38055800

[17] Valentine M, Wilson D, Gresnigt MS, Hube B. Vaginal Candida albicans infections: host-pathogen-microbiome interactions. FEMS Microbiol Rev. 2025;49:fuaf013. 10.1093/femsre/fuaf013 PMC12071381 40347186

[18] Citiulo F, Jacobsen ID, Miramón P, Schild L, Brunke S, Zipfel P, et al. Candida albicans scavenges host zinc via Pra1 during endothelial invasion. PLoS Pathog. 2012;8(6):e1002777. 10.1371/journal.ppat.1002777 PMC3386192 22761575

[19] Mayer FL, Wilson D, Hube B. Candida albicans pathogenicity mechanisms. Virulence. 2013;4(2):119. 10.4161/viru.22913 PMC3654610 23302789

